# Escherichia Coli: an Important Pathogen in Patients with Hematologic Malignancies

**DOI:** 10.4084/MJHID.2014.068

**Published:** 2014-11-01

**Authors:** Daniel Olson, Abraham T. Yacoub, Anita D. Gjini, Gelenis Domingo, John N. Greene

**Affiliations:** 1 University of South Florida Morsani College of Medicine. 12901 Bruce B Downs Blvd, Tampa, FL 33612; 2 H. Lee Moffitt Cancer Center and Research Institute. 12902 Magnolia Drive. Tampa, Florida 33612-9497; 3 Moffitt Cancer Center and Research Institute. 12902 Magnolia Drive. Tampa, FL 33612-9497

## Abstract

**Background:**

Escherichia coli (E. coli) is a pathogen of great concern in immunosuppressed patients. While antimicrobial prophylactic therapy has become the standard, the emergence of resistant pathogens has some questioning its use. This study describes our experience with E.coli as a pathogen in neutropenic patients with a hematologic malignancy, and addresses future directions of treatment for this patient population.

**Methods:**

A retrospective chart review of 245 E.coli bacteremia patients at Moffitt Cancer Center from 05/18/02 – 05/15/12 was conducted. Out of 245 patients, 169 did not meet the criteria due to non-neutropenic status, or not diagnosed with a hematologic malignancy, or due to having insufficient medical records. Thus, they were excluded from the study. As a result, 76 patients were involved in this study. Patients were identified via microbiology laboratory computerized records.

**Results:**

The included patients experienced clinically significant E.coli bacteremia resulting in a median hospital stay of 14.7 days. Several patients developed severe sepsis requiring the use of pressor and ventilator therapy.

**Conclusions:**

E.coli is a major pathogen in these patient populations resulting in extended hospital stays and specialized treatment to overcome their E.coli bacteremia. The data supports the use of fluoroquinolone prophylactic therapy, however, earlier detection and treatment of neutropenic infection is needed.

## Introduction

Neutropenia is a blood disorder characterized by an abnormally low number of white blood cells called neutrophils. Neutrophils are essential in the defense against bacterial and fungal pathogens, thus neutropenic patients are highly susceptible to these pathogens.^1^ Hematological cancer patients are already at an increased risk of infection due to chemotherapy-induced immunosuppression.^2^ Therefore, when patients with hematologic malignancies become neutropenic, they are at an even higher risk for developing infectious complications.

These infections can result in significant morbidity and mortality due to the development of febrile neutropenia and bacteremia.^3^

Several organisms are responsible for causing infection in hematologic patients with neutropenia. Various fungi, as well as, Gram-negative and Gram-positive bacteria were found to be causes of infection in neutropenic patients with hematological malignancies.^4^

In recent years, *E.coli* sequence type ST131 has been given much worldwide attention as an emerging multi-drug resistant (MDR) pathogen. Data suggests that this sequence of *E.coli* may be the main explanation for recent increases in antimicrobial resistance prevalence in E.coli.^7^ Serious extra-intestinal infections with this MDR *E.coli* ST131 often leave physicians with limited treatment options, higher costs, and increased usage of “last resort” antimicrobials, such as carbapenems.^7^

The use of antimicrobial prophylaxis in neutropenic patients has shown some effect in reducing infectious complications. In particular, fluoroquinolones, are widely used to protect patients against Gram-negative bacteremia.^8^ However, the use of these drugs has to be weighed against the emerging possibility of producing antibiotic-resistant bacterial strains such as *E.coli* ST131.^2^ Still, the true impact of fluoroquinolone prophylaxis in regards to treatment efficacy and adverse effects is only partially known.^2^

The next frontier for treating neutropenic patients with hematologic malignancies may deal with monitoring microbial gut diversity through treatment. As part of their treatment regimens, such patients may undergo hematopoietic stem cell transplantation (HSCT), also known as bone marrow transplantation (BMT). They are exposed to chemotherapy, radiation, and antimicrobials in a short time period as part of their treatment.^9^ As a result, the equilibrium between the intestinal microbiota and mucosal epithelium is disrupted, causing large shifts in bacterial populations inhabiting the gut thus making the patient susceptible to bloodstream infections.^9^ While studies have assessed that this microbial shift does occur, we have yet to find data answering the following question. Can monitoring these microbial shifts help us treat, or even prevent, MDR *E.coli* bacteremia infections in neutropenic patients with hematologic malignancies?

## Methods

This study used a retrospective chart review design and was approved by the IRB prior to data collection and analysis. A data list was obtained from Moffitt Cancer Center records containing the names of all *E.coli* bacteremia patients from 05/18/02 – 05/15/12. Patients were identified through review of Moffitt’s institutional databases: Cerner/PowerChart and Emageon at Moffitt Cancer Center. Patients were included in the study if they met the following criteria: at least 18 years of age, diagnosed with hematological malignancy, and neutropenic at time of E.coli bacteremia (defined as absolute neutrophil count < 500 cells/μL).

Using the *E.coli* bacteremia data list obtained from Moffitt Cancer Center records, 245 *E.coli* bacteremia patients were examined for possibility of study inclusion. Out of the 245 original patients, 169 did not meet the inclusion criteria, and thus were excluded from the study. Of the 169 excluded patients, 40 patients were not neutropenic at time of positive *E.coli* blood culture, 119 patients did not have a hematologic malignancy diagnosis, 9 patients had insufficient records, and 1 patient was found to not have *E.coli* bacteremia. Therefore, 76 patients met eligibility and are included in the data analysis. Two patients developed multiple neutropenic *E.coli* bacteremia episodes separated by at least 6 months, which were treated as separate subject events for data analysis. If any patient produced multiple positive *E.coli* blood cultures within the same month, the earliest culture was used for study analysis.

The survival rate post *E.coli* bacteremia was measured in order to evaluate the long-term prognosis of these patients. Patient records were used to determine how many days each patient lived after their positive *E.coli* blood culture. A Kaplan-Meier survival curve was generated to show survival at various time intervals.

Information on all study patients was stored in a password-protected database, maintained by investigators at Moffitt Cancer center. Patients’ data was kept on file until the regulated time (per IRB requirements). We then compiled the research findings in an excel file that contained the medical record numbers (MRNs). Upon completion of data analysis, all direct identifiers (e.g. MRN, DOB, etc) were removed from the Excel sheet. All files are password protected and only accessed by the research team. No paper records were kept for this study and no patient identifiers were disclosed to anyone besides the investigative team.

## Results

### Patient Demographics

Of the 76 subjects included in the data analysis, 23 had undergone a BMT at the time of their *E.coli* bacteremia, whereas 53 had not. The BMT-Group patients ranged from 35–75 with a median age of 60 years, and the Non-BMT patients ranged from 22–82 years with a median age of 55. Males outnumbered females in both groups, making up 73.91% of the BMT-Group, and 62.26 % of the Non-BMT Group. Of the 23 patients in the BMT-Group, 15 had undergone an autologous BMT, whereas 8 had undergone an allogenic BMT.

Regarding hematologic malignancies, the BMT-Group had 12% Acute myeloid leukemia (AML), 12% Acute lymphocytic (or lymphoblastic) leukemia (ALL), 4% Chronic myelogenous leukemia (CML), 8% Hodgkin’s lymphoma (HL), 24% Non-Hodgkin’s lymphoma (NHL), 28% Multiple myeloma (MM), 8% AML + myelodysplastic syndrome (MDS), 4% HL + NHL, and 0% ALL+ CML. In comparison, the Non-BMT Group had 13% ALL, 33% AML, 2% CML, 0% HD, 26% NHL, 7% MM, 2% MDS, 7% AML + MDS, 2% ALL + CML, 0% HD + NHL, and 7% with some other hematologic malignancy.

### Severity of E. coli bacteremia infection

Each patient was classified as having bacteremia without septic syndrome, having sepsis, severe sepsis, or septic shock as outlined by current clinical practice definitions.^10^ Several parameters were also used to assess the overall severity of the *E.coli* bacteremia infections in each group. These parameters included: hospital stay length in days when *E.coli* bacteremia occurred, days of neutropenia at time of positive *E.coli* blood culture, whether patient was placed on pressor therapy, whether patient was placed on ventilator, and whether patient required hemodialysis.

**Figure 1:**
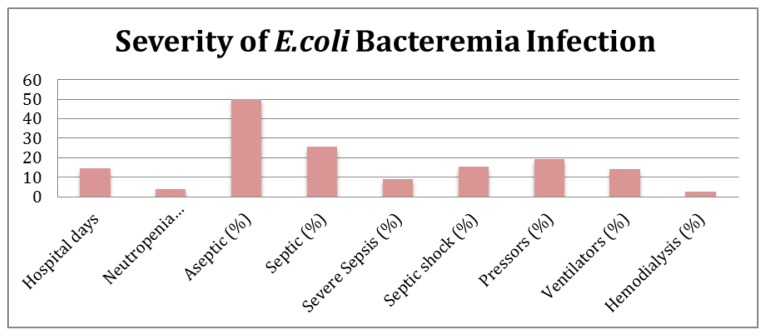


The following results were yielded when looking at the total patient population. The median hospital stay was 14.7 days; while the median length of neutropenia at time of positive *E.coli* blood culture was 4 days. In terms of bacteremia severity, 50% were classified as aseptic, 26% were septic, 9% were severe sepsis, and 15% were in septic shock. In addition, pressor treatment was required in 19%, ventilator treatment was required in 14%, and hemodialysis was required in 2% (Figure 1, Table 1).

### Survival after E. coli Bacteremia

**Figure 2:**
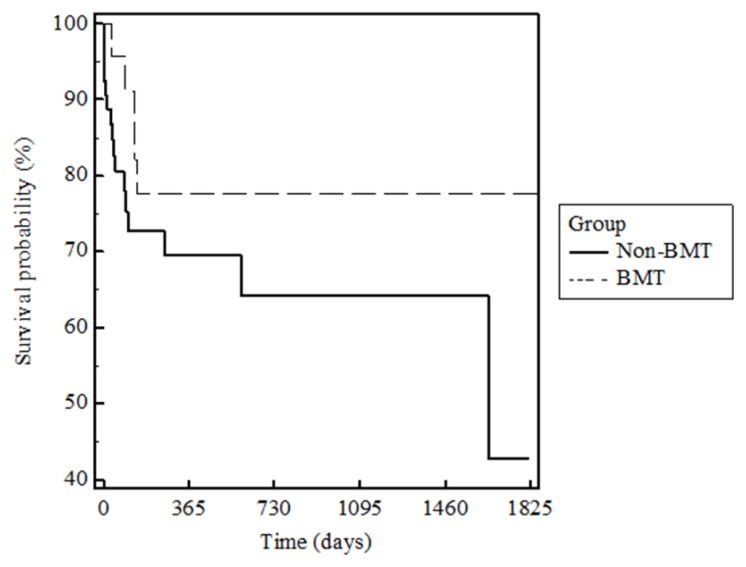
Patient Survival Rates

The survival curve showed that the BMT-group patients had better survival outcomes in both the short and long-term when compared to the non-BMT group (Figure 2).

### E. coli Antibiotic Resistance

**Figure 3:**
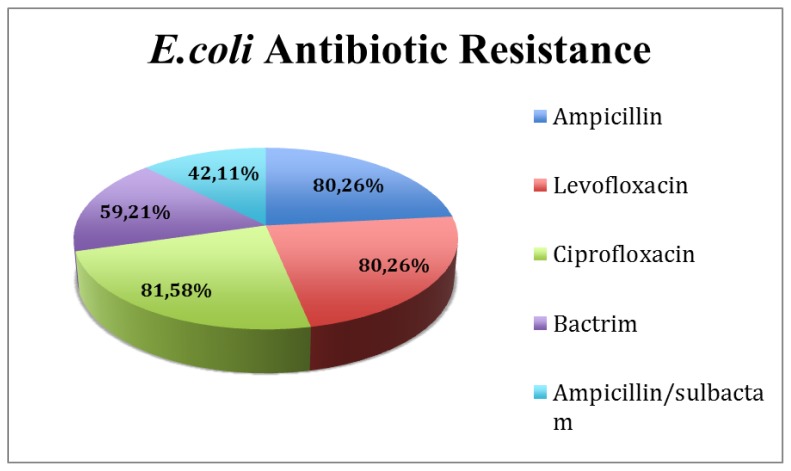


**Figure 4:**
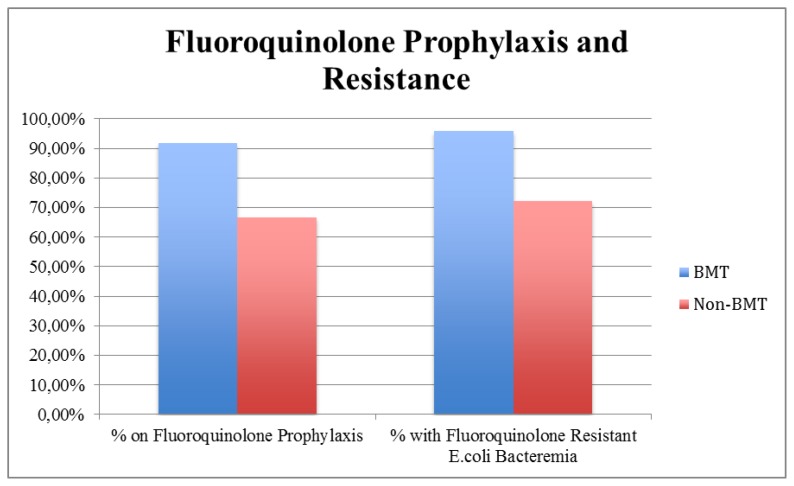


Antibiotic resistance was measured using the *E.coli* bacteremia microbiology reports obtained from PowerChart at Moffitt Cancer Center. The top 5 antibiotics the *E.coli* was resistant to were: ampicillin, ciprofloxacin, levofloxacin, trimethoprim and sulfamethoxazole, and ampicillin/sulbactam. Of the total patient population, 81.58% were resistant to ciprofloxacin, 80.26% were resistant to levofloxacin, 80.26% were resistant to ampicillin, 59.21% were resistant to trimethoprim and sulfamethoxazole, and 42.11% were resistant to ampicillin/sulbactam (Figure 3). The BMT-Group had 91.67% of its patients on a fluoroquinolone prophylaxis, with 95.83% of patients developing fluoroquinolone resistant *E.coli* bacteremia, whereas the No-BMT group had fewer patients, 66.67%, on fluoroquinolone prophylaxis, with 72.22% developing fluoroquinolone resistant *E.coli* bacteremia (Table 2, Figure 4).

## Discussion

We support that fluoroquinolone resistance in this population is a growing problem. The etiology of bacteria in the fecal flora changes dramatically after quinolone prophylaxis.^11^ Existing studies have shown that fluoroquinolone prophylaxis in hematologic cancer patients has been linked to an increase the incidence of Gram-negative fluoroquinolone resistant bacteria such as E. Coli. Quinolone resistant *E. Coli* have caused breakthrough bacteremia during prophylaxis with quinolones.^11^ However, it has been stated that this increase in antibiotic resistant bacteria is not necessarily responsible for increased morbidity.^2^ Our study found similar results. The BMT-Group had a much higher percentage of its subjects on a fluoroquinolone prophylactic therapy than the non-BMT group. However, the BMT-Group had less hospital days and shorter neutropenia duration at time of positive *E.coli* blood culture than the Non-BMT group. Also, the BMT-Group patients had less severe *E.coli* bacteremias as measured by our aseptic, septic, severe sepsis, and septic shock categories. Thus, the data supports the claims made by existing studies, in that the higher occurrence of fluoroquinolone resistant bacteria does not always mean increased morbidity.^2^

We support the use of fluoroquinolone prophylaxis for neutropenic patients with hematologic malignancies. If patients develop an *E.coli* bacteremia while on these drugs, there is a higher chance of the *E.coli* being fluoroquinolone resistant. Previous analysis demonstrated that *E. Coli* resistance to fluoroquinolones is significantly related to previous prophylaxis with these agents, also that is closely associated with extended-spectrum-B-lactamase (ESBL) production. This finding could suggest a potential indirect influence of fluoroquinolones resistance on clinical outcome in hematological cancer patients, related to ESBL production.^12^ However, fluoroquinolone resistance was not associated with worse outcomes in this study. In addition to the use of quinolone prophylaxis, we feel there needs to be a better way of effectively adjusting treatment regimens for neutropenic infections. The current practice of changing antimicrobials when the patient develops neutropenic fever puts the patient at a high risk for developing serious bacteremia infections.

Prophylactic regimens of quinolones may predispose the patient to dangerous bloodstream infections with multi-drug resistant pathogens such as *E.coli* ST131^7^ or ESBL-producing *E. Coli* strains. Another concern is the rate of relapsing bacteremia in patients who are on treatment for a hematological malignancy and also on fluoroquinilone prophylaxis. Gram-negative bacteria are significantly more frequent among relapsing bacteremia compared to non-relapsing cases and this phenomenon may be due to an imbalance of enteric microflora induced by fluoroquinolone prophylaxis.^13^ Cattaneo et al demonstrated that ESBL-producing *E. Coli* was foundto be present in more then a 25 percent of cases at the first episode of bacteremia and at relapses. Fluoroquinolone resistance was recorded in all episodes of relapsing *E. Coli* bacteremia.^13^ Instead of changing their prophylactic antimicrobials when they develop neutropenic fever, perhaps we can get ahead of the game. Recent studies have shown the ability to monitor microbial gut diversity in allogenic-HSCT patients throughout treatment via fecal sampling methods.^9^ This monitoring was able to predict which patients were more prone to develop bloodstream infections as a result of intestinal domination by certain toxins.^9^ Perhaps these methods can be applied to the treatment of this population. Being able to identify infection with dangerous strains of microbes, such as *E.coli* ST131, may allow us to adjust antimicrobial treatment before serious infectious complications can occur. In doing so, we may be able to more effectively treat, or even prevent serious *E.coli* bacteremia infections that are experienced in this subject population.

The Kaplan-Meier survival analysis showed that the BMT group had better short-term as well as long-term survival than the non-BMT group. This data does not support the suggestion from previous studies that neutropenia in BMT patients is associated with higher mortality when compared to neutropenia in Non-BMT patients.^4^ In regards to BMT therapy, the data is positive. It shows that undergoing a BMT procedure may not put the patient at risk for worse outcomes regarding neutropenic *E.coli* bacteremia. We understand that many factors were not controlled for when comparing these two patient groups, thus further research needs to be done to evaluate survival differences.

We realize that our study is limited in various ways. The data could potentially be limited by the relatively small sample size as well as taking place at a single institution. Also, it was unfeasible for us to control for the individual differences in the physicians that treated these subject patients. We acknowledge that our study was limited to a relatively small group of patients. Further research should attempt to achieve larger subject populations at multiple institutions to fully assess *E.coli* bacteremia infections in this patient population.

## Conclusion

In summary, *E.coli* is a major pathogen in hematologic malignancy patients with neutropenia. Patients in this population can experience extended hospital stays and specialized treatment to overcome their, often serious, *E.coli* bacteremia infections. We support the use of fluoroquinolones in neutropenic patients with hematologic malignancies. These drugs were associated with a higher chance of the *E.coli* being fluoroquinolone resistant; however, this was not associated with poorer outcomes.

Still, further research needs to be done to improve treatment of neutropenic infection in this patient population. Current treatment guidelines leave patients at risk for developing potentially serious bacteremia infections. We believe that future research should examine the efficacy of fecal microbiota monitoring to adjust treatment guidelines. Perhaps these methods will one day allow us to prevent serious *E.coli* bacteremia infections in this patient population.

## Figures and Tables

**Table 1:** Severity of E.coli Bacteremia Infection

Variable	
Hospital days	14.7
Neutropenia days at +cx	4
Aseptic (%)	50
Septic (%)	25.64
Severe Sepsis (%)	8.97
Septic Shock (%)	15.38
Pressors (%)	19.23
Ventilators (%)	14.1
Hemodialysis (%)	2.56

**Table 2:** Fluoroquinolone Prophylaxis and Resistance by Group

Group		Patients with Fluoroquinolone Resistant *E.coli* Bacteremia
BMT	91.67%	95.83%
Non-BMT	66.67%	72.22%

## References

[1] Palumbo A, Blade J, Boccadoro M (2012). How to Manage Neutropenia in Multiple Myeloma. Clinl Lymphoma Myeloma Leuk.

[2] Cattaneo C, Quaresmini G, Casari S (2008). Recent changes in bacterial epidemiology and the emergence of fluoroquinolone-resistant Escherichia coli among patients with haematological malignancies: results of a prospective study on 823 patients at a single institution. Journal of antimicrobial chemotherapy.

[3] Gafter-Gvili A, Paul M, Fraser A (2007). Antibiotic prophylaxis in neutropenic patients. IMAJ-RAMAT GAN.

[4] Collin BA, Leather HL, Wingard JR (2001). Evolution, incidence, and susceptibility of bacterial bloodstream isolates from 519 bone marrow transplant patients. Clin Infect Dis.

[5] Nosanchuk J, Sepkowitz K, Pearse R (1996). Infectious complications of autologous bone marrow and peripheral stem cell transplantation for refractory leukemia and lymphoma. Bone Marrow Transplant.

[6] Bames RA, Stallard N (2001). Severe infections after bone marrow transplantation. Curr Opin Crit Care.

[7] Johnson JR, Johnston B, Clabots C (2010). Escherichia coli sequence type ST131 as the major cause of serious multidrug-resistant E. coli infections in the United States. Clinical Infect Dis.

[8] Reuter S, Kern WV, Sigge A (2005). Impact of fluoroquinolone prophylaxis on reduced infection-related mortality among patients with neutropenia and hematologic malignancies. Clin Infect Dis.

[9] Taur Y, Xavier JB, Lipuma L (2012). Intestinal Domination and the Risk of Bacteremia in Patients Undergoing Allogeneic Hematopoietic Stem Cell Transplantation. Clin Infect Dis.

[10] Lever A (2007). Sepsis: definition, epidemiology, and diagnosis. BMJ.

[11] Chong Y, Shimoda S, Yaushiji H (2014). Clinical Impact of Fluoroquinolone-Resistant Escherichia coli in the Fecal Flora of Hematological Patients with Neutropenia and Levofloxacin Prophylaxis. PLoS One.

[12] Trecarichi EM, Tumbarello M, Spanu T (2009). Incidence and clinical impact of extended-spectrum-beta-lactamase (ESBL) production and fluoroquinolone resistance in bloodstream infections caused by Escherichia coli in patients with hematological malignancies. J Infect.

[13] Cattaneo C, Antoniazzi F, Tumbarello M (2014). Relapsing bloodstream infections during treatment of acute leukemia. Ann Hematol.

